# Outcome of the First Electron Microscopy Validation Task Force Meeting

**DOI:** 10.1016/j.str.2011.12.014

**Published:** 2012-02-08

**Authors:** Richard Henderson, Andrej Sali, Matthew L. Baker, Bridget Carragher, Batsal Devkota, Kenneth H. Downing, Edward H. Egelman, Zukang Feng, Joachim Frank, Nikolaus Grigorieff, Wen Jiang, Steven J. Ludtke, Ohad Medalia, Pawel A. Penczek, Peter B. Rosenthal, Michael G. Rossmann, Michael F. Schmid, Gunnar F. Schröder, Alasdair C. Steven, David L. Stokes, John D. Westbrook, Willy Wriggers, Huanwang Yang, Jasmine Young, Helen M. Berman, Wah Chiu, Gerard J. Kleywegt, Catherine L. Lawson

**Affiliations:** 1 MRC Laboratory of Molecular Biology, Hills Road, Cambridge CB2 0QH, UK; 2 Department of Bioengineering and Therapeutic Sciences, Department of Pharmaceutical Chemistry, California Institute for Quantitative Biosciences, Byers Hall Room 503B, University of California at San Francisco, 1700 4th Street, San Francisco, CA 94158, USA; 3 National Center for Macromolecular Imaging, Verna and Marrs McLean Department of Biochemistry and Molecular Biology, Baylor College of Medicine, Houston, TX 70030, USA; 4 National Resource for Automated Molecular Microscopy, Department of Cell Biology, The Scripps Research Institute, La Jolla, CA 92037, USA; 5 Department of Chemistry and Chemical Biology and Research Collaboratory for Structural Bioinformatics, Rutgers, The State University of New Jersey, 610 Taylor Road, Piscataway, NJ 08854, USA; 6 Life Sciences Division, Lawrence Berkeley National Laboratory, University of California, Berkeley, CA 94720, USA; 7 Department of Biochemistry and Molecular Genetics, University of Virginia, Charlottesville, VA 22904, USA; 8 Howard Hughes Medical Institute, Department of Biochemistry and Molecular Biophysics, Columbia University, 650 West 168th Street, New York, NY 10032, USA; 9 Department of Biological Sciences, Columbia University, 1212 Amsterdam Avenue, New York, NY 10027, USA; 10 Rosenstiel Basic Medical Sciences Research Center and Howard Hughes Medical Institute, Brandeis University, MS 029, Waltham, MA 02454, USA; 11 Markey Center for Structural Biology and Department of Biological Sciences, Purdue University, West Lafayette, IN 47907, USA; 12 Department of Life Sciences and the National Institute for Biotechnology in the Negev, Ben-Gurion University of the Negev, Beer-Sheva 84105, Israel; 13 Department of Biochemistry and Molecular Biology, The University of Texas, Houston Medical School, Houston, TX 77030, USA; 14 Division of Physical Biochemistry, MRC National Institute for Medical Research, London NW7 1AA, UK; 15 Department of Biological Sciences, Purdue University, 240 S. Martin Jischke Drive, West Lafayette, IN 47907, USA; 16 Institute of Complex Systems (ICS-6), Forschungszentrum Jülich, 52425 Jülich, Germany; 17 Laboratory of Structural Biology Research, National Institute of Arthritis and Musculoskeletal and Skin Diseases, NIH, Building 50, Room 1517, 50 South Drive, MSC 8025, Bethesda, MD 20892, USA; 18 Skirball Institute of Biomolecular Medicine, New York University School of Medicine, New York, NY 10012, USA; 19 D. E. Shaw Research, 39th Floor, 120 W. 45th Street, New York, NY 10036, USA; 20 Protein Data Bank in Europe, EMBL-EBI, Wellcome Trust Genome Campus, Hinxton, Cambridge CB10 1SD, UK

## Abstract

This Meeting Review describes the proceedings and conclusions from the inaugural meeting of the Electron Microscopy Validation Task Force organized by the Unified Data Resource for 3DEM (http://www.emdatabank.org) and held at Rutgers University in New Brunswick, NJ on September 28 and 29, 2010. At the workshop, a group of scientists involved in collecting electron microscopy data, using the data to determine three-dimensional electron microscopy (3DEM) density maps, and building molecular models into the maps explored how to assess maps, models, and other data that are deposited into the Electron Microscopy Data Bank and Protein Data Bank public data archives. The specific recommendations resulting from the workshop aim to increase the impact of 3DEM in biology and medicine.

## Main Text

### Introduction: Background and Goals of the Meeting

#### 3DEM and Molecular Modeling Based on 3DEM Data Are Well-Established

Structure analysis of macromolecular complexes using three-dimensional electron microscopy (3DEM) has become an essential tool for structural biology research. 3DEM is uniquely able to determine the structural organization of macromolecular complexes not amenable to other methods (Frank, 2006; Glaeser et al., 2007). More than thirty years ago, low-dose imaging and computational averaging of images of two-dimensional (2D) crystals of bacteriorhodopsin produced a density map that revealed protein α helices spanning the lipid bilayer (Henderson and Unwin, 1975). Subsequent advances in 3DEM of unstained specimens embedded in vitreous ice (cryo-EM) are increasingly yielding density maps of a wide variety of specimens at near-atomic resolution. Applications to icosahedral viruses and chaperonins already demonstrate that 3DEM maps can be good enough to trace Cα backbones de novo and to visualize some side-chain densities without the aid of X-ray crystallography (Chen et al., 2011; Liu et al., 2010; Zhang et al., 2010a, 2010b).

3DEM is unusually versatile and can be used to investigate the structures of a wide variety of specimens under conditions close to those in the cell. Specimens can range from highly purified, homogeneous molecular complexes to heterogeneous conformations and may assume different forms with or without symmetry. Subnanometer resolution cryo-EM structures are found to be increasingly useful in providing illustrative snapshots of macromolecular machines such as the ribosome, chaperonins, and viruses bound to various cellular effectors or ligands (Becker et al., 2009; Frank et al., 1995; Miyazawa et al., 2003; Zhang et al., 2010b). Finally, electron tomography, in which a series of images is collected from a region of the specimen tilted to different viewing angles, can be used to obtain 3D density maps of individual macromolecular particles, including pleiomorphic ones for which whole-particle averaging is inadmissible (Grünewald et al., 2003), as well as sections, or even whole cells, provided they are not thicker than approximately 0.7 μm (Al-Amoudi et al., 2004, 2007; Frank, 2006; McIntosh, 2007; Medalia et al., 2002). For an extensive review of 3DEM procedures, see Baker and Henderson (2012).

Interpretation of a 3DEM density map frequently involves building a molecular model. Models may consist of atoms or “coarse-grained” objects representing multiple atoms, such as whole residues, secondary structure segments, and shape-based features. A model of a given macromolecular complex is often computed by assembling experimentally determined atomic structures or homology models of the individual subunits. The subunit models can either be held rigid (Chapman, 1995; Jiang et al., 2001; Lasker et al., 2009; Roseman, 2000; Rossmann, 2000; Volkmann and Hanein, 1999; Wriggers et al., 1999; Wriggers and Chacón, 2001) or allowed to flex (Fabiola and Chapman, 2005; Rusu et al., 2008; Schröder et al., 2007; Tama et al., 2004; Topf et al., 2005, 2008; Trabuco et al., 2008; Trabuco et al., 2011; Wriggers et al., 2000; Zhang et al., 2011) while being fit into the map; precautions need to be taken to avoid over-fitting by introducing too many refinable parameters relative to the data available. At higher resolutions (better than 6 Å for a mostly α-helical structure or 4 Å for a mostly β-stranded structure), it may be possible to recognize known folds of protein subunits (Jiang et al., 2001; Khayat et al., 2010; Saha et al., 2010). In addition to density map features and protein stereochemistry, modeling may also utilize other types of information, such as symmetry, protein proximities from proteomics experiments, residue proximities from chemical cross-linking, related homologous structures, and SAXS profiles (Alber et al., 2008).

**Figure 1:**
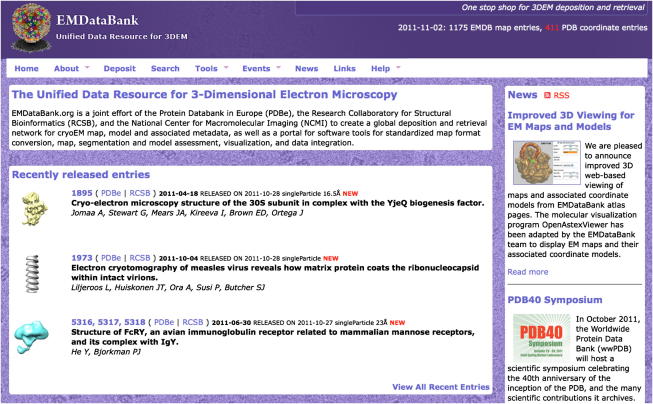
EMDataBank, Unified Data Resource for 3DEM Home Page Available at http://emdatabank.org.

**Figure 2:**
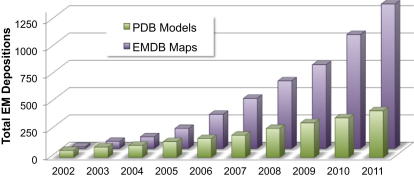
3DEM Entries in EMDB and PDB, Cumulative by Year Statistics for December 31, 2011: 1322 map entries, 427 model entries.

Increasingly, 3DEM maps and models described in the literature are deposited in public archives, where they can be retrieved for independent assessment, use, and development of new tools for visualization, fitting, and validation. EMDataBank, the Unified Data Resource for 3DEM (http://emdatabank.org; Lawson et al., 2011; Figure 1), provides joint deposition and retrieval of maps in the Electron Microscopy Data Bank (EMDB) archive as well as coordinates of the models fitted into map volumes in the Protein Data Bank (PDB) archive. Currently, more than 1,000 EM maps and more than 400 map-derived models are available (Figure 2).

#### 3DEM Maps and Models Need to Be Validated

Every 3DEM map and model has some uncertainty. Therefore, an assessment of map and model errors is essential, especially when a wide range of techniques are used by a variety of practitioners. In addition, as with all rapidly developing fields, in our enthusiasm to go further and faster, there is a risk that avoidable mistakes, both large and small, may be made in the production or interpretation of maps. Such mistakes may have the adverse effect of undermining the credibility of 3DEM methods in general. It is therefore important to develop methods for checking our conclusions and validating maps and models, with the goal of establishing a set of best practices for the field.

Historically, the 3DEM field has not made any notorious blunders but, as with all scientific disciplines, a handful of papers have reported erroneous results. While the rarity of these incidents is heartening, they do provide good justification for being cautious. In the early days of electron crystallography, the resolution of published projection maps was sometimes overly optimistic (Hayward and Stroud, 1981) before the importance of correcting for beam tilt was realized (Henderson et al., 1986). It has also proved remarkably easy to get the absolute hand wrong even in subnanometer resolution structures (Böttcher et al., 1997; Kühlbrandt and Wang, 1991; Li et al., 1997; Zhou et al., 2000). Images of tilted and untilted specimens together provide all the information needed to correctly determine this property (Belnap et al., 1997; Cheng et al., 2002; Conway et al., 1997).

In the single particle electron microscopy field, five papers between 2002 and 2005 independently reported different structures of the same receptor complex, the 1.3 MDa inositol phosphate receptor, a tetramer responsible for calcium release from the endoplasmic reticulum. Two of the structures were determined in negative stain and three in amorphous ice (da Fonseca et al., 2003; Hamada et al., 2003; Jiang et al., 2002; Sato et al., 2004; Serysheva et al., 2005). Although the differences between the maps may be partly explained by differences in biochemical preparation, they are more likely due to errors in the structure determination. A more recent cryo-EM study at ∼10 Å (Ludtke et al., 2011b), while being substantially different from the three earlier cryo-EM maps, agrees qualitatively with one of the negative stain structures (Hamada et al., 2003), so there is evidence of convergence. Although each of these studies used methods that were the best available at the time, the absence of appropriate validation tools has meant that it was not possible either to prove the structures were correct or to show they were incorrect.

Additionally, it is common practice to fit crystal structures or homology models into cryo-EM maps. A discrepancy in model-based interpretation of the 2.5 MDa ryanodine receptor remains unresolved and illustrates the challenge in fitting molecular fragments into low-resolution maps (Serysheva et al., 2008; Tung et al., 2010). In the earlier study, a homology model was docked into the map region indicated by antibody labeling, while in the other study, global fitting with an X-ray structure of a fragment was performed. These differences in the protocol were sufficient to completely alter the final model and illustrate the need to not only identify the best-fitting location for a fragment and determine a confidence interval, but to also consider possible conformational variability of the fragment being docked.

Given the current rapid increase in the size, productivity, and impact of the 3DEM community, it is timely to suggest guidelines for validating, annotating, and depositing 3DEM maps and map-derived models. There is an opportunity to synergize experimental and computational efforts by bringing together the respective communities. There is a need to establish standards as well as to share software and databases. Similar efforts in the X-ray crystallography (Read et al., 2011), nuclear magnetic resonance (NMR) spectroscopy (http://www.wwpdb.org/workshop/2010/nmr_validation.html), and modeling communities (Schwede et al., 2009) can serve as constructive examples.

#### Meeting Aims

Twenty eight participants from 19 academic institutions worldwide attended a meeting at Rutgers on September 28 and 29, 2010 (http://vtf.emdatabank.org). The participants discussed issues in the computation and validation of 3DEM maps and models as well as ways to strengthen the collaboration between the experimental and modeling communities. The participants' consensus was formulated as specific recommendations, aimed to increase the impact of 3DEM in biology and medicine.

#### Meeting Program

On the first day, 14 presentations focused on computing maps from raw 3DEM data, and computing molecular models from maps were given. On the second day, independent “map” and “model” discussion groups were asked to address specific questions related to the deposition and validation of 3DEM maps and models, respectively, report on their findings, and make recommendations for the future. These two discussion groups are referred to as the Map Group and the Model Group respectively throughout the rest of this review.

We now summarize the consensus of recommendations reached among the participants of the meeting. The recommendations are concerned with derivation, annotation, archiving, visualization, distribution, and publication of EM maps and models based on the maps. These recommendations are intended to be a starting point for further refinement by a broader community of scientists interested in EM.

### Recommendations by the Map Group

The Map Group's discussion began by enumerating the attributes of a 3DEM map, among the most important of which are the method used to prepare the sample and the symmetry of the object examined.

The sample can be prepared in many different ways, but here we describe the four most frequently used techniques. First, negatively stained samples are usually prepared by adsorption onto a carbon film, followed by washing with a few drops of negative stain, such as 1% uranyl acetate, and then dried, resulting in images where the molecules of interest are seen as low-density regions from which the stain has been excluded. Second, ice-embedded samples are normally prepared by blotting a thin film of a solution containing the molecules of interest, then plunge-freezing the film into liquid ethane at a temperature just above its freezing point (Dobro et al., 2010; Dubochet et al., 1988). The images in this case show the structures as regions of higher density against a background of vitreous ice. Third, samples, often 2D crystals, can also be deposited on a continuous carbon film and embedded in ice or a medium other than ice or negative stain, such as glucose, trehalose, or tannic acid (Unwin and Henderson, 1975). This treatment frequently preserves the high-resolution diffraction order, but contrast matching at low resolution can obscure the molecular envelope. Finally, sections of tissue or other specimens can be prepared either by plastic embedding (Glauert and Lewis, 1998) or high pressure freezing and cryo-sectioning (Ladinsky, 2010), followed by tomographic data collection and 3D structure determination. Plastic embedding requires care in interpretation due to possible fixation artifacts, and cryo-sectioning can produce compression artifacts; nonetheless, both are widely used and valuable methods.

The nature of the maps that are computed from 2D EM images depends principally on the symmetry of the objects being examined. Thus, there are maps for 2D crystal structures that can be obtained using either crystallographic methods (Henderson et al., 1986) or single-particle approaches (Frank et al., 1988); maps for helical structures that can be obtained using either Fourier-Bessel methods (Diaz et al., 2010) or single-particle approaches (Egelman, 2010); single-particle maps, including structures with icosahedral symmetry, other point group symmetries or no symmetry (Rochat and Chiu, 2012); and finally tomogram and sub-tomogram average maps (Schmid and Booth, 2008).

#### Standards for Assessing Resolution and Accuracy of Maps Need to Be Developed

It is clear that the community needs validation methods for assessing the accuracy of 3DEM maps. A satisfactory validation method does not yet exist, and its development remains an open research problem. However, there are a number of conditions that are necessary for map validity, as well as some methods that may detect whether a map is incorrect under certain circumstances. The majority of these methods require at least a 3D reconstruction without any post-processing, such as masking or filtration. Most of these methods also require access to some portion of the raw data and metadata used to produce the reconstruction. A few methods require collection of additional data explicitly for validation. Examples of some validation methods are given below.

##### Absolute Hand Determination

The absolute hand of a structure cannot be determined without either a tilt experiment or sufficient resolution to resolve chiral features directly in the map. Tilt experiments also offer the opportunity to validate the accuracy of the structure as a whole and can help place limits on orientation accuracy. Such methods include random-conical tilt (Radermacher, 1988), orthogonal tilt (Leschziner and Nogales, 2006), single-particle tomography (Baumeister et al., 1999), and tilt-pair parameter plots (Henderson et al., 2011; Rosenthal and Henderson, 2003) for which a web-based service is available (https://cryoem.nimr.mrc.ac.uk/software/). The absolute hand can often be established by comparison of the structures of component subunits whose hands have been determined previously. The availability of structures in a number of such subunits, or subunit domains within a complex, can validate or correct the hand determination (Baker et al., 2003; Kanamaru et al., 2002; Leiman et al., 2004). In addition, the hand of an icosahedral capsid with chiral surface lattice (such as T = 7l) can be easily distinguished at low resolution by the arrangement of hexameric capsomeres in images of freeze-fracture, metal shadowed particles (Prasad et al., 1993).

##### Data Coverage and Agreement between Raw Images and Class Averages

Additional validation methods used in single-particle reconstruction include ensuring agreement between projections of the 3D structure and raw images or (if generated) class-averages, ensuring that reference-free class-averages are fully represented among the set of model projections, and ensuring sufficient coverage of particle orientations (Orlova et al., 1996; Tang et al., 2007). These criteria represent necessary but not sufficient criteria for a reliable 3D reconstruction.

##### Statistical Assessment of the Map

Map variance and local resolution determination, such as bootstrap-based variance maps (Penczek et al., 2006) and local Fourier Shell Correlation (FSC) measurements (Ménétret et al., 2007), can provide additional measures to help interpret structures. For maps with resolution better than 20 Å by the 0.5 FSC criterion, RMEASURE (Sousa and Grigorieff, 2007) can be used to estimate resolution and signal-to-noise directly from the map based on correlation of neighboring Fourier Transform terms. Possible bias from a starting model or overfitting of noise should also be estimated and statistics provided where possible.

##### Recommendation

Experimentalists should be encouraged to assess their own maps according to the criteria listed above and report the methods that they used when depositing the maps. To help the community as a whole, EMDataBank should develop a table of existing map validation techniques with a description of what experimental data are required for each technique, the circumstances under which the technique can be used, the software package(s) (with links) that implement the technique, and what aspect of the reconstruction the technique validates. This table can be updated as new methods are developed. Any published method should be considered as a candidate for inclusion in the list, as the list would not mandate any tests to be performed, but simply present possible tools available for authors to validate their own data. As methods are tested on a variety of data sets, the field will begin to establish which techniques are reliable for each specific task. Additionally, EMDataBank should gather and provide raw benchmark data that would enable the community to test the methods.

#### Map Resolution Should Be Reported, and Visible Structural Features Should Be in Accordance with the Claimed Resolution

The single-particle map resolution is typically evaluated according to the FSC of two maps constructed from independent data sets (Penczek, 2010b). However, the threshold value for the map resolution has not been uniformly reported (Rosenthal and Henderson, 2003). Because of various experimental and computational factors that lead to damping of the Fourier amplitudes of the images, the final density map needs to be scaled to retrieve the detailed features (Böttcher et al., 1997). One method is to apply a Gaussian function equivalent to a temperature factor in X-ray crystallography (Fernández et al., 2008; Rosenthal and Henderson, 2003); another method is to apply a one-dimensional structure factor obtained from the X-ray scattering or a model (Baker et al., 2010; Gabashvili et al., 2000; Penczek, 2010a). At resolution better than 4.5 Å, the helical pitch and β strand separation should be visible in addition to some bulky side-chains. At subnanometer resolution, secondary structure features, such as long α helices and large β sheets, should begin to emerge. Several existing tools allow quantitative assessment of secondary structure (Baker et al., 2007; Kong et al., 2004). If the level of secondary structure visible in the map does not agree with the cited resolution and the sequence-based structure prediction, the resolution estimate may not be accurate. At lower resolution (10–20 Å), the situation is more complex and requires more care. In some favorable situations, domain or molecular boundaries may be delineated. At still lower resolution (>20 Å), a simple point-spread function plot may be adequate, along with a statement of the RMS noise level, estimated from presumed featureless regions (Fan and Ellisman, 2000).

##### Recommendation

Deposition of a published map should include its full FSC curve to the Nyquist frequency on a linear spatial-frequency scale (Frank, 2006, see pages 250 and 251). If the final experimental volume was masked in any way, FSC curves should be provided for both the masked and unmasked versions. If the half-datasets compared in FSC were separated at the outset of analysis, this should be stated; otherwise, it will be assumed that a less independent comparison in which the whole data set was aligned against a common model was performed. Intermediate levels of independence between the gold standard of carrying out two completely independent analyses and common 3D model throughout should also be described. Reconstruction software packages should include the option to produce an unmasked map after the last cycle of refinement.

Map manipulations and transformations other than magnification and contrast transfer function correction should also be reported. Examples include density stretching (e.g., negative density truncation), high- and low-pass filtering, sharpening, signal-to-noise ratio weighting, thresholding, damping (e.g., FOM weighting), cropping, and masking. The key parameters in different reconstruction algorithms should be reported. In addition, the quantitative assessment of the map features, such as segmentation and feature extraction, should be included in the deposition.

#### Map Symmetry Should Be Validated

Many 3DEM specimens are composed of multiple copies of the same proteins, and thus, symmetry may exist in the complex. However, the symmetry may break down for different functional states. Reconstruction may be carried out with or without symmetry imposed; this treatment needs to be explicitly reported and justified.

##### Recommendation

A program is required to read in the density map, recognize any point group, helical or translational symmetry, and print out the point group or helical or space group symmetry, and the precise origin. This processing would ensure that the stated symmetry is real and that the stated origin is correct. The program would ideally also provide the transformation to reorient a map into the standard coordinate frame for the point group or helical symmetry (Heymann et al., 2005; Lawson et al., 2008).

#### Map Depositions Should Include Annotations Specific to Each Map Type

3DEM map types can be classified as 2D crystal, helical array, single-particle, tomogram, and sub-tomogram average. The reconstruction algorithms are unique to each of these specimens/map types, and thus the corresponding annotations are unique.

##### Recommendation

For each specimen/map category, there should be clear definitions for what data are deposited, and these are outlined as follows.

##### 2D Crystal Maps

The following data should be deposited: structure factor file following X-ray crystallographic conventions and including the space group, symmetry applied, raw intensities (I), and error estimates (σI) if electron diffraction data are available; amplitudes (A), phases (φ), σA, σφ if only image data are available; map A, φ, and figure of merit if both diffraction data and image data are available; any information about twinning if present; and a 3D map. There should be an option to deposit merged A, φ lists, such as those produced by LATLINE (Agard, 1983). If the 2D images have been processed using single-particle methods, the single-particle deposition procedure can be used (below). In addition, point group symmetries should be indicated when they exist, including cyclic C_n_ and/or dihedral D_n_ symmetries.

##### Helical Maps

Helical filaments and tubes are currently being reconstructed by Fourier-Bessel and single-particle approaches with different data requirements for deposition. In both cases, a helically-symmetric 3D volume needs to be submitted, and the orientation of the helical symmetry axis must be noted as well as the coordinates of this axis. For the Fourier-Bessel methods, the layer line data decomposed into complex G(R) functions (containing amplitudes and phases as a function of the distance R from the meridian of the transform [Klug et al., 1958]) should be deposited in one of two forms. The first form is the conventional form of G_n,l_(R), where n is the Bessel order and l is the layer line number. The helical repeat c needs to be entered in Å, and the units/turn (u/t) should be entered as a ratio of two integers. The second form is the more general form of G_n,Z_(R), where n is the Bessel order and Z is the spacing (in Å^−1^ from the equator). The axial rise per subunit should be entered (in Å), and the u/t should be entered as a real number. The convention is that negative values of n correspond to left-handed helices. For the single-particle approaches, the screw symmetry (axial rise in Å and rotation in degrees per subunit) must be described, with the convention that negative angles correspond to a left-handed helix. The same resolution and Eulerian angle statistics as proposed for single particles below should be deposited.

##### Single-Particle Maps

Single-particle maps include maps with icosahedral, other point group symmetries, and no symmetry. For maps derived from single-particle reconstructions, the following additional information should be supplied: the quality of the raw data (experimental B-factor of the raw images and data processing); methods for initial map generation and for iterative map refinement, including full description of any imposed or no symmetry; particle orientation (Eulerian angle) distribution coverage; statistical confidence of the map (variance map); methods of map sharpening, masking, and filtering; FSC curves or other resolution estimates; and handedness determination method. If the deposited map has been manually masked by the authors, both the unmasked and masked maps should be deposited. In addition, validation statistics from the raw map produced by the reconstruction software should also be provided. If the map has been segmented, the segmentation should be deposited. If extra averaging has been done within an asymmetric unit (e.g., for T = 13 icosahedral symmetry), the fully averaged sub-volume should be deposited. Other useful data include the power spectrum inside and outside a soft mask as well as raw tilt pair images and tilt pair parameter plots.

##### Tomograms

The tomographic approach is especially well-suited for studying pleomorphic single particles of biological assemblies, organelles, and whole cells. The resolution of a tomogram is difficult to assess, though a quantitative measure of the alignment of images in a tilted series can be determined. RMS deviation of fiducial gold particles between frames in a tomographic series is one good indicator for tomogram quality. The deposition should include the respective reconstructions from the tilt series, and, if available, segmentation volumes/masks. The tilt series angular coverage and spacing, the name of the reconstruction algorithm, and estimates of the alignment error, such as fiducial marker RMS information, should also be deposited. An estimate of the reconstruction resolution, such as that obtained from the noise-compensated leave-one-out method, where (e.g., FSC = 0.5) resolution is plotted against the tilt angle of the frame (Cardone et al., 2005) and an estimate of the non-isotropic point-spread function showing the expected vertical and in-plane resolution, should also be included. Better measures of resolution and reliability need to be developed.

##### Sub-Tomogram Averages

Sub-tomogram extraction, sorting, aligning, and averaging has become a routine approach to determine 3D structures of conformationally identical components at a higher resolution. It is imperative that a standard assessment in terms of resolution and map reliability is developed. The deposited data should include the final averaged map that appeared in the publication. Validation statistics should be generated for the raw averaged volume created from the sub-volumes without filtration or masking. The total number of sub-volumes/particles should be stated, along with an FSC curve for the sub-volumes used, and the tilt series angular coverage and spacing should be provided. Finally, the reconstruction algorithms that were used to create the tomogram, to align it in 3D, to classify the sub-volumes, and to create the average should be identified.

#### The Requirements and Practicalities for the Archiving of Raw 3DEM Data Files Should Be Investigated

Raw 2D images and raw unmasked maps are needed for many validation processes, as described above. There is mixed opinion on the archiving of the raw images because of the size and the logistics of the data transfer and storage. For example, a single set of raw single-particle EM particle image data might contain 20,000 particles in 500 × 500 boxes, totaling 5GB, using a byte image format. A raw cryo-EM tomographic series might contain 70 frames of 2K × 2K pixels, so 280 MB; a complete tomogram would be 2K × 2K × 256, so 1–3 GB. Furthermore, to make such data meaningful, the metadata that describe important microscope parameters, such as the microscope model used, kV, Cs, Cc, aperture sizes, illumination conditions, energy filter settings, magnification, defocus, astigmatism, beam tilt if determined, beam convergence/divergence, and tilt angles, must be defined.

##### Recommendation

Tools operating on raw data should be made available to individual laboratories to enable reporting of validation results. EMDataBank should investigate the practicalities of archiving raw images and describe the metadata as well as the storage capabilities that would be required.

While it is currently impractical to archive raw images from all publicly archived EM reconstructions due to size (e.g. > 1TB for some projects), we recommend that a portal be established to archive selected raw image datasets. Since several labs have already made their raw data publicly accessible, the portal can simply serve as a pointer to those sites. The availability of raw image datasets will facilitate development of improved image-processing procedures (LeBarron et al., 2008; Shaikh et al., 2008) as well as improved molecular modeling algorithms.

### Recommendations by the Modeling Group

The Modeling Group discussion began by enumerating four important attributes of macromolecular assembly models based on EM data.

First, representations of a model with different degrees of granularity can be used. Each “particle” in a model may represent an atom, a side-chain centroid, a small contiguous cluster of atoms, a domain, or even a whole protein (Alber et al., 2008). In addition, secondary structure elements (Baker et al., 2007) and segments of the map (Pintilie et al., 2010; Wriggers et al., 1998, 2010) can be represented by a variety of geometrical objects. Representations other than those using one particle per atom are frequently referred to as coarse-grained, reduced, or multi-scale (Grubisic et al., 2010; Kolinski, 2011).

Second, the degrees of freedom explored in a search for a model that best fits a map can vary. The explored degrees of freedom depend on the representation and can be further limited by the sampling algorithm. For example, in rigid body fitting, only the position and orientation of the subunit model are computed, but in flexible fitting, the model conformation as well as position and orientation are computed. For maps with sufficiently high resolution, de novo models can be generated. The distinction between flexible, rigid body, and de novo fitting is important for assessing the signal-to-noise ratio and/or data-to-parameter ratio as well as for distinguishing between the precision (variability among the well-scoring models) and accuracy (closeness to the truth) of a fitted model. The number of refinable parameters and available data need to be considered to avoid over-fitting.

Third, different types of information in addition to the 3DEM map can be used to augment the generation of a model. For example, a subunit model can be derived either completely (in rigid fitting) or partly (in flexible fitting) by other means, including X-ray crystallography NMR spectroscopy, and with a lesser accuracy by SAXS measurement, comparative modeling, and ab initio structure prediction. Moreover, the relationships between subunits can be informed by complementary data, such as proteomics experiments, chemical cross-linking, a related homologous assembly structure, or a SAXS profile (Lasker et al., 2010; Robinson et al., 2007).

Fourth, either a single set of model coordinates or an ensemble of model coordinates (as is frequently the case for reporting NMR-based structures) can be produced to reflect the ambiguity in the coordinates, given the input data. Frequently, the variability in the ensemble is meant to represent the precision of the model and represents the lower bound on the error. As an alternative to an ensemble of models, the precision can be represented, for example, by variation for each coordinate in a single model.

These four attributes can, in principle, vary across different parts of the model. It should ideally be possible to provide a hierarchical representation (i.e., multiple representations applied to the same component of the assembly) as well as different representations for different components. For example, one subunit may be determined at high resolution while another subunit could be represented as a sphere if no atomic structure is known.

#### Criteria for Assessing Models Must Be Established

The usefulness of a model strongly depends on its accuracy; different applications that use the models have varied requirements for model accuracy and precision. As with structural models derived by other techniques, accuracy can be estimated globally for the whole model or locally for each specific part (e.g., residue). There are three sets of fundamentally different criteria for assessing a model based on a 3DEM map, all of which should generally be satisfied.

First, the conformation of a subunit and interfaces between subunits can be assessed without regard to the 3DEM map. The corresponding criteria for assessment of the internal consistency of a model with known molecular constraints (e.g., on geometry, conformation, and molecular interactions) include those proposed by the PDB working groups focused on assessment of crystallographic (Read et al., 2011), NMR (http://www.wwpdb.org/workshop/2010/nmr_validation.html), and modeled (Schwede et al., 2009) structures.

Second, a model can be assessed with regard to the 3DEM map. A sample set of corresponding criteria for agreement of the model with the map are produced by the EMFIT program (Rossmann, 2000, 2001), including atomic clashes, component interactions, chemical properties, fit to the map, as well as a composite criterion that quantifies model quality relative to a background distribution. Other programs that provide statistical measures for assessing a model in the context of a 3DEM map include CoAn (Volkmann, 2009) and E2HSTAT (available in EMAN2, Ludtke et al., 1999). A correlation coefficient between a map determined by EM and a map calculated from a model can also be used (Jiang et al., 2001; Pintilie et al., 2010; Wriggers and Chacón, 2001), as can residue-based and overall real-space R values (Brändén and Jones, 1990). Comparisons of the cross-correlation to other metrics, such as those borrowing from machine learning techniques, enable systematic and objective evaluation of scoring functions (Vasishtan and Topf, 2011). More studies on the evaluation tools themselves are needed.

Finally, a model can be assessed with regard to additional data about the structure that were not used in model calculation. Such data may include cross-linking, antibody labeling, sites of specific labels (such as carbohydrate moieties), proximity of known features to recognizable positions in the map, chemical properties consistent with the environment, and spectroscopic measurements.

Assessment criteria should be as independent as possible from the objective function that is optimized during fitting (Kleywegt, 2009). At low resolution, a large number of non-EM-derived constraints are typically used in model construction, potentially reducing the informative value of certain assessment criteria. For example, analysis of the main-chain stereochemistry of a rigid-body fitted structure has no bearing on the accuracy and quality of the obtained model, but rather reflects the quality of the high-resolution structure that was used to fit the low-resolution data. Consideration of the modeling and fitting procedures is therefore an important component of the assessment.

Methods for estimating model accuracy are being developed; no accurate or dominant method has yet emerged. There is a great need to assess the model quality based on the data-to-parameter ratio and precision, but anecdotal evidence suggests that such methods are not yet reliable. Approaches that begin to address this issue include cross-validation (Schröder et al., 2010) and quantifying the best-fitting model relative to alternative fits (Tung et al., 2010; Vasishtan and Topf, 2011; Volkmann and Hanein, 1999; Volkmann, 2009; Wriggers and Birmanns, 2001) or the fitting to the mirror image map (Rossmann, 2000). The predicted accuracy should depend on the map variance. In assessing the quality of a map, all of these criteria need to be satisfied within reasonable tolerance. The EMFIT program copes with this problem by taking the average of each attribute expressed as the number of standard deviations above the mean of random fits (Rossmann et al., 2001). It also needs to be determined whether or not a map computed from a flexibly fitted model fits within the error bars of the original map equally well as the original model (if it does, there is no information in the map to justify flexible fitting). The Bayesian inferential structure determination approach originally proposed for NMR structure determination (Rieping et al., 2005) could also be applied to EM-based modeling. Finally, accuracy measures that convey the suitability of models for specific applications need to be established.

##### Recommendation

We recommend coordinated development of model assessment criteria and corresponding software, with special emphasis on criteria reflecting the suitability of models for specific end-user applications. EMDataBank should provide a technical platform to make validated tools for estimating model accuracy available to the users of the models; it should also establish a mechanism for continuous evaluation and improvement of these tools.

#### Community-wide Benchmarks for Modeling Methods Need to Be Created

With a growing number of density maps and models, it is urgent that a clear set of standards be established for benchmarking existing and new modeling techniques. In addition to a publically available benchmark data set, it will also be necessary to establish an open community forum to report the algorithm and results used in the various modeling approaches.

##### Recommendation

To facilitate the development and appreciation of quality criteria as well as development of methods for modeling based on 3DEM maps in the first place, we recommend establishment of community-wide benchmarks of cases with experimentally determined 3DEM maps and known structures (e.g., from X-ray crystallography). Then, the correlation between quality criteria and actual geometrical accuracy can be determined empirically. The Cryo-EM Modeling Challenge effort (Ludtke et al., 2011a) will be helpful in this regard, as well as establishing a prototype modeling web-portal. If possible, these benchmark cases should also include “raw” micrograph (particle) images.

#### Sequences of All Components Need to Be Annotated

##### Recommendation

We recommend that both “biological” and modeled subunit sequences be clearly defined in map-derived models. Even when the biological sequence is known (e.g., it may be preferred to fit an X-ray structure of a homolog instead of a homology model of the “biological” sequence when the confidence in a homology model is lower than in the X-ray structure), deposition of a subunit model with the sequence different from the biological sequence should be allowed. Finally, if known, subunit sequences should be listed for a 3DEM map even if there is not a molecular model for them.

#### Capability to Archive Coarse-Grained Representations of Models Is Needed

As discussed, not all 3DEM models contain atomistic representations of proteins. In some cases, such as de novo models in which Cα backbone traces are constructed, one point or pseudoatom may represent an entire amino acid residue. In other instances, pseudoatoms may be used for flexible fitting of coarse-grained features or simple geometrical description of secondary structure, or even a whole protein subunit can be used in annotating the macromolecular structure.

##### Recommendation

We recommend that PDB consider generalizing the representations of molecular models that can be deposited to account for the variety of possible model representations. Ideally, a general representation scheme and the corresponding file format would be used for derivation, annotation, archiving, visualization, distribution, and publication of models.

#### Standards for Data Formats Must Be Established to Facilitate Data and Software Exchange

Although the crystallographic and NMR spectroscopy communities have essentially reached a consensus on the definition of common data formats that enable the seamless exchange of data and algorithms (Westbrook and Fitzgerald, 2003; Winn, 2003), most software tools for building models based on 3DEM maps use proprietary data formats for input data, parameters, and results. Although data formats from experimental structures can be applied to the protein model coordinates, data types specific to 3DEM-based modeling and specific details of the individual modeling algorithms frequently vary between different applications. This incompatibility is a serious impediment to the exchange of tools and algorithms; it hinders both method development and the widespread use of tools outside of the developer groups themselves.

##### Recommendation

We recommend that EMDataBank initiate a community-wide mechanism for reaching an agreement on a common open data format for information related to molecular modeling based on 3DEM data with the aim of facilitating the exchange of algorithms and data.

### General Recommendations

#### Journals Should Encourage Map and/or Model Deposition before Publication

At the present time, models are published with widely varying levels of descriptive information about how they were derived. A set of guidelines for what should be included in a paper needs to be established. These guidelines should be shared with journal editors and reviewers. We encourage journals to require proof of map and/or model deposition before publication.

Models that have been peer reviewed and referred to in published literature should be publicly available. Without access to the model coordinates and sufficient annotation of the model, it is impossible for the reader to evaluate the results and to assess the validity of published interpretations.

##### Recommendation

We recommend that EMDataBank, in collaboration with the 3DEM community, suggest standards for journal publication, define minimum annotation standards, and establish the scope and requirements of a public archive of models based on EM maps.

#### EMDataBank Can Play a Key Role

The discussion at the Workshop explored how to maximize the impact of the 3DEM public data archives.

##### Recommendation

We recommend that EMDataBank consortium members work together with the 3DEM community to provide unified access to molecular models and their annotations and support the development of data standards to facilitate exchange of information and algorithms. EMDataBank should play an active role in facilitating discussions about data standards between developers of computational methods and their users, provide access to tools for estimating model accuracy, and promote their further development. Its user interface should allow a broad range of queries to the model database as well as links to experimental data. Tools for estimating model errors and selecting the likely best model among the available models should be included. An interface to interactive model evaluation services should be established. Mechanisms to notify users when a particular sequence is modeled (or experimental data becomes available) should be implemented. The EMDataBank consortium should work to establish a series of online documents with community feedback to explain the value and limitations of protein structure models based on EM data. The 3DEM public data archives should be as inclusive of all method developers and modeling methods as technically feasible.
